# Patterns-of-Life Aided Authentication

**DOI:** 10.3390/s16101574

**Published:** 2016-09-23

**Authors:** Nan Zhao, Aifeng Ren, Zhiya Zhang, Tianqiao Zhu, Masood Ur Rehman, Xiaodong Yang, Fangming Hu

**Affiliations:** 1 School of Electronic Engineering, Xidian University, Xi’an 710071, China; nan_zhao_@hotmail.com (N.Z.); afren@mail.xidian.edu.cn (A.R.); zhiyazhang@163.com (Z.Z.); tqzhu@mail.xidian.edu.cn (T.Z.); fangming95@163.com (F.H.); 2 Centre for Wireless Research, University of Bedfordshire, Luton LU1 3JU, UK; Masood.UrRehman@beds.ac.uk

**Keywords:** Wireless Body Area Networks, initial trust, patterns-of-life aided authentication

## Abstract

Wireless Body Area Network (WBAN) applications have grown immensely in the past few years. However, security and privacy of the user are two major obstacles in their development. The complex and very sensitive nature of the body-mounted sensors means the traditional network layer security arrangements are not sufficient to employ their full potential, and novel solutions are necessary. In contrast, security methods based on physical layers tend to be more suitable and have simple requirements. The problem of initial trust needs to be addressed as a prelude to the physical layer security key arrangement. This paper proposes a patterns-of-life aided authentication model to solve this issue. The model employs the wireless channel fingerprint created by the user’s behavior characterization. The performance of the proposed model is established through experimental measurements at 2.45 GHz. Experimental results show that high correlation values of 0.852 to 0.959 with the habitual action of the user in different scenarios can be used for auxiliary identity authentication, which is a scalable result for future studies.

## 1. Introduction

Recent years have seen a massive development of wireless sensors. This has enabled Wireless Body Area Networks (WBANs)/Wireless Body Sensor Networks (WBSNs) to become a key technology in real-time health monitoring of patients, providing effective detection and treatment of acute diseases [1,2,3,4,5,6,7]. It is envisioned that WBAN/WBSN will have an annual device shipment of 187.2 million units by 2020 [2,3]. The WBANs/WBSNs have successfully proceeded through the adoption phase devising efficient and flexible prototyping and management [8,9]. However, the potential of the WBANs/WBSNs is severely daunted by the challenges of security and privacy of the user′s important personal information [10,11,12,13]. WBAN nodes need to be simple in hardware and interface due to form-factor, size and energy limitations. This decreases the scope of the traditional non-password authentication mechanisms that mostly require advanced hardware or major modifications to the system software [14]. On the other hand, Physical Layer Security (PLS) schemes are well suited in this scenario. This is due to the fact that the distance between two body-mounted sensor nodes is expected to be much smaller than the distance between a body-mounted sensor and an eavesdropping node in the patient-monitoring WBAN applications. This results in a maximum ambiguity to the eavesdropping party [15]. Although PLS key generation can ensure secrecy, it requires the node authentication as a prelude [16]. Therefore, establishing initial trust (iTrust) between the authentication node (AN) and wearable node (WN) acts as the fundamental step towards a WBAN physical layer security protocol.

Researchers have proposed various techniques in the literature to use the PLS for security and authentication [17,18,19]. Physical authentication through the actual positioning in wireless local area network is employed in [17]. Indoor space channel detection and ray-tracing tools are used to achieve authentication in [18] while identification and verification of the sender is attained through channel vector in [19]. Key distribution in WBANs has also been dealt in different ways [20,21]. Human body movement-aided authentication is also considered in [22,23] but the focus is on treating the human body motion as the source of jamming.

This study attempts to provide a novel solution to this problem using the patterns-of-life aided authentication model (PLAM) employing the wireless channel features to quantify a user’s behavior habits as an authentication parameter. The wireless channel fingerprint formed by the characterization of user’s behavior depicts the correlation between the AN and the WN. The WN is worn by the user while performing his daily life actions. The AN, placed at different positions in the vicinity, interacts with the WN automatically and generates a life pattern fingerprint for identification. This eradicates the need of a pre-distributed secure key and danger of lost key and a failed traditional authentication mechanism in the event of a stolen sensor node [14]. To the best of the authors’ knowledge, this technique is novel and such a method is not being reported in the open literature.

Following the introduction, this paper is organized in four sections. Section 2 describes the experimental set-up to generate user’s patterns-of-life data and presents measured data. Section 3 provides correlation analysis of AN and WN for establishing initial trust and authentication based on the observed patterns-of life. Conclusions and potential future work are provided in Section 4.

## 2. Experimental Set-up

This work considers the iTrust (initial-Trust) establishment in a WBAN where the user has worn the WN. The user could be stationary or mobile.

**Figure 1:**
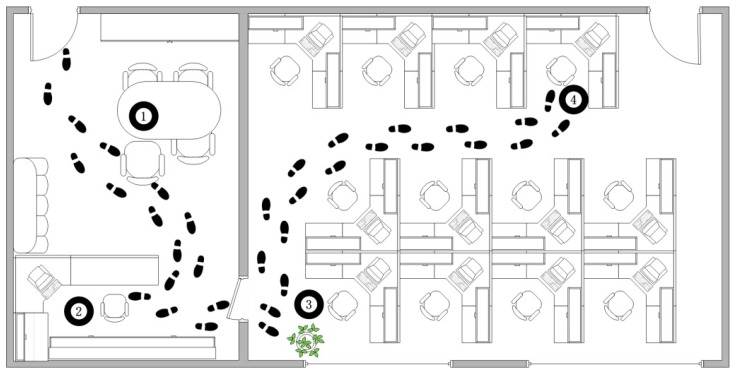
Experiment site map.

**Figure 2:**
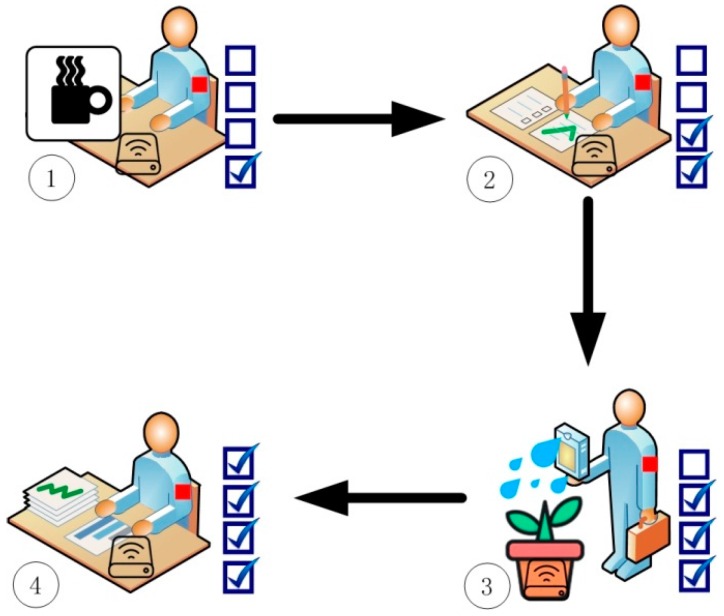
Considered user actions of (**1**) eating breakfast; (**2**) sign in to work; (**3**) watering the flowers and (**4**) go into operation.

**Figure 3:**
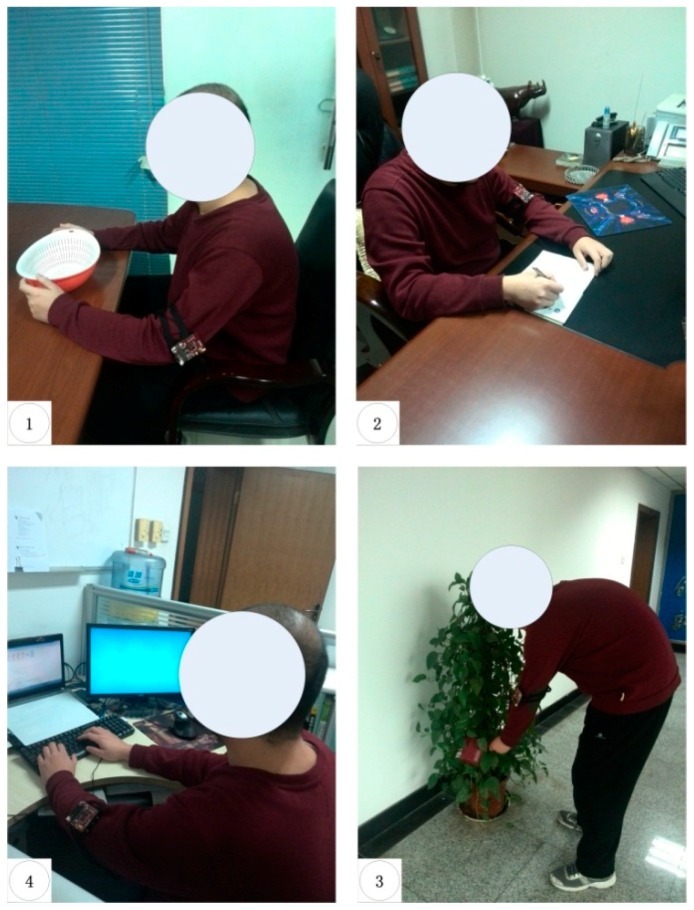
Photos for four scenarios explained in Figure 2.

The measurements were carried out in a typical laboratory environment with dimensions of H × L × W = 3 m × 7.8 m × 10.8 m inside the new science and technology building at Xidian University. Four ANs installed in the tea table, attendance book, flowerpot and workshop were considered. Figure 1 shows the experimental setup with the ANs being represented by the numbers 1, 2, 3, and 4. The footprints illustrate the daily movement pattern of the user between the four spots. To prove the idea, a common experimental approach is adopted by taking the channel measurements while the user is performing four habitual actions. The daily life actions considered include (1) eating breakfast; (2) sign in to work; (3) watering the flowers and (4) go into operation, as shown in Figure 2 and Figure 3. The users had the liberty to choose their pattern of the four activities initially. This pattern is termed as “template” and was being used for pre-registration. It forms the iTrust value for the ANs. The user is required to repeat the behavioral pattern maintaining the same sequence to ensure the authentication. Hence, if the wireless channel fingerprint of the user′s behavior characterization between the AN and WN is found to be in line with the PLAM, the WN obtain an iTrust value is depicted as a check mark in Figure 2. Any drift from the behavioral pattern would result in a failed authentication.

**Figure 4:**
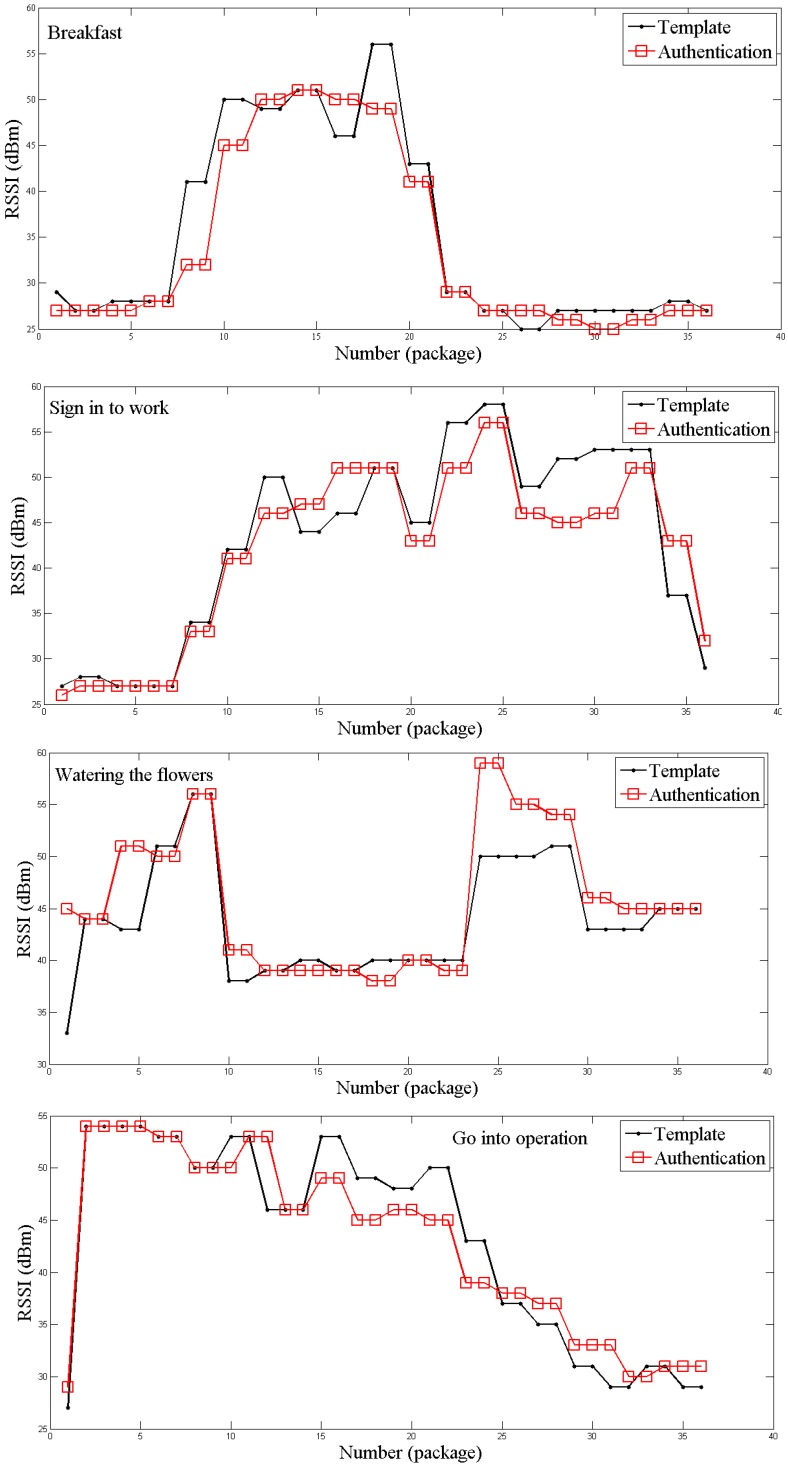
Comparison of measured initial trust (iTrust) and authentication data.

The WBANs are no longer based on stand-alone devices but an integration of various portable electronics operating in the vicinity of the human body. To represent this integrated WBAN environment, the measurements were performed using HBE-Ubi sensor node in half-duplex, two packets per second mode. It sends and receives 36 packages and calculates received signal strength indicator (RSSI) as follows:

(1) $$RSSI=P_{TX}\left(dBm\right)-PL\left(dBm\right)$$

(2) $$AN_{RSSI}=WN_{RSSI}+SI+PoL$$

where $P_{TX}\left(dBm\right)$ is transmit power, PL(dBm) represents path loss, SI is surrounding influence and PoL takes into account patterns-of-life radio frequency loss, respectively. The initial behavioral pattern forms the fingerprint template while the successive actions need to follow this template in order to attain iTrust. The comparison of the template and the pattern of the user’s successive movements is presented in Figure 4. The results show meaningful strong correlation between PLAM fingerprint (black line) and the user’s movements (red line).

## 3. Analysis and Discussion

Measured data is used to establish correlation between the PLAM fingerprint and the user′s movements in the four considered scenarios. Correlation coefficient values for the four configurations are given in Table 1. These results indicate that the high correlation between the two configurations can well serve the need of iTrust and authentication. As this study adopts a proof-of-concept approach, the measurement data sampling has been limited which can be extended further in practical implementations. It is, however, evident from the analysis of the measured data that even by using simple correlation, enough resolution has been attained to achieve identity authentication.

**Figure 5:**
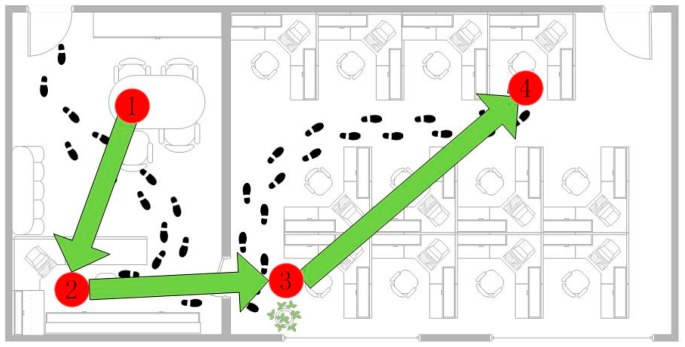
iTrust fingerprint according to user’s behavior characterization.

Figure 5 highlights the daily routine of the user obtained through the measurements. It is clear that the users were first eating breakfast, and then signing-in to work followed by watering the flowers and going into operation. The results also show that in this PLAM, a correlation coefficient value of ≥0.85 between the AN and WN will enable the AN to obtain the iTrust value.

The priori knowledge available to us in the PLS is that the distance between the nodes is greater than half a wavelength. The characteristics of the RSSI sequence make the measurements inaccurate if measured at a distance greater than the half wavelength. Therefore, the channel statistics can be regarded as independent of the distance. It implies that in a practical scenario, the attacker Eve cannot measure the channel between the legitimate users unless she intrudes into the house and successfully imitates the legitimate user’s “pattern-of-life” (which is a far-fetched possibility). Hence, PLAM ensures the authentication. 

Although, the experiment adopts a simple approach with four representative daily life scenes to establish the usability of the technique, the use of channel characterization of human behavior to achieve identity authentication can be easily expanded to any scene. Moreover, this technique consumes no additional packages. Authentication is extracted from the RSSI sequence of packets required for normal communication between the WBAN nodes such as time alignment, power monitoring, etc. In fact, the proposed technique does not require any communication packet but achieve authentication through the observation of the changes in the channel conditions due to the interaction between the human body and the surrounding environment.

The results of these initial experiments clearly show that the use of the PLAM in the WBAN applications provides a viable authentication solution. The results presented in this paper are in-line with the foundings of other researchers where the human body movement pattern is considered as unique, stable and distinguishable to be used for the node authentication [23,24,25]. Wang et al. have considered the effects of body motion as a noise into the system [23]. Wu et al. have considered the authentication between the body-mounted nodes, which are not more than 0.2 m apart [24]. In [25], locking/unlocking a smart phone by a specific hand waving pattern is evaluated. However, as the presented technique is a novel approach in the WBANs and no such authentication method has been reported, we cannot provide a performance comparison at this point.

## 4. Conclusions

Establishing an initial trust between the WBAN nodes is a prelude to the physical layer authentication method. This paper has presented a method of establishing initial trust in the WBAN nodes by characterization of human behavior and its comparison with the PLAM fingerprint. The study has shown that the initial trust with user’s patterns-of-life can be achieved that can reduce the consumption of communication packages to minimum which is much valuable for the resource constrained WBAN systems. A preliminary PLAM model has been developed to realize auxiliary status recognition using patterns-of-life through multiple authentication nodes acquiring initial trust value. The working of the model has been evaluated through measurements providing a preliminary implementation of the PLAM multi-node initial trust identification using a HBE-Ubi-Sensor node module. Experimental results show that users can obtain a high correlation value of 0.852 to 0.959 by following a set pattern for their daily activities in different scenarios enabling authentication and effectively removing contingency, which is a scalable result.

Future work will focus on the PLAM fingerprint optimization and iTrust establishment without use of a template. Moreover, proving security against a reasonable adversarial model is also an aspect of future work.

## Figures and Tables

**Table 1:** Summary of correlation coefficients for the received signal strength indicator (RSSI) sequences in four measurement scenarios.

Scenario	Correlation Coefficient
Eating breakfast	0.959
Sign in to work	0.943
Watering the flowers	0.852
Go into operation	0.963
